# Penicillin-binding proteins exhibit functional redundancy during asymmetric cell division in *Clostridioides difficile*

**DOI:** 10.1128/jb.00503-25

**Published:** 2025-11-26

**Authors:** Shailab Shrestha, Gregory A. Harrison, Jules M. Dressler, Morgan E. McNellis, Aimee Shen

**Affiliations:** 1 Department of Molecular Biology and Microbiology, Tufts University School of Medicine 12261, Boston, Massachusetts, USA; 2 Program in Molecular Microbiology, Tufts University Graduate School of Biomedical Sciences 50910, Boston, Massachusetts, USA; University of Notre Dame, Notre Dame, Indiana, USA

**Keywords:** asymmetric division, penicillin-binding proteins, functional redundancy, sporulation, *Clostrioides difficile*

## Abstract

Peptidoglycan synthesis is an essential driver of bacterial growth and division. The final steps of this crucial process involve the polymerization of glycan strands by shape, elongation, division, and sporulation (SEDS) family glycosyltransferases and the cross-linking of peptide cross-bridges by class B penicillin-binding proteins (bPBP). While many bacteria use distinct bPBPs to perform specialized roles during a given cellular process, some bPBPs can play redundant roles, particularly in the presence of certain cell wall stresses. Our understanding of these compensatory mechanisms, however, remains incomplete. Endospore-forming bacteria typically encode multiple bPBPs to drive morphological changes required for sporulation. The sporulation-specific bPBP, SpoVD, synthesizes the polar division septum and the cortex peptidoglycan layer during sporulation in the pathogen *Clostridioides difficile*. Although SpoVD catalytic activity is essential for cortex synthesis, we show that it is partially dispensable for asymmetric division. The dispensability of SpoVD’s catalytic activity requires the presence of its SEDS partner, SpoVE, and another sporulation-induced bPBP, PBP3. While PBP3 localizes to the polar septum and interacts with components of the polar division machinery, the ability of PBP3 to promote division during sporulation occurs independent of its catalytic activity. Notably, this latter finding differs from previously reported modes of functional redundancy in bacteria, indicating that there are diverse mechanisms by which penicillin-binding proteins can be functionally redundant in bacteria.

**IMPORTANCE:**

Peptidoglycan synthesis requires the transpeptidase activity of penicillin-binding proteins (PBPs), which have specialized functions during cell growth, division, and differentiation. However, many bacteria produce PBPs with overlapping functions, and this functional redundancy can lead to increased antibiotic resistance. While the major pathogen, *Clostridioides difficile*, requires the SpoVD PBP to form spores, we found that its transpeptidase activity is dispensable for asymmetric division, the first morphological stage of sporulation, because a sporulation-induced PBP, PBP3, partially substitutes for SpoVD’s function during this stage. Since PBP3’s ability to promote asymmetric division in this context does not depend on the its catalytic activity, unlike prior studies of PBP functional redundancy, our analyses highlight the diversity in mechanisms used to enable functional redundancy between PBPs.

## INTRODUCTION

Peptidoglycan (PG) synthesis is an essential driver of the morphological changes required for bacterial growth and division. This crucial process is driven by the glycosyltransfer reactions that polymerize the glycan strands and the transpeptidation reactions that cross-link the peptide side chains between the strands (1, 2). These enzymatic reactions require the activities of high-molecular-weight (HMW) penicillin-binding proteins (PBPs), which are divided into two classes based on their catalytic ability: class A PBPs (aPBP) are bifunctional enzymes capable of both glycosyltransferase and transpeptidase activities, while class B penicillin-binding proteins (bPBPs) are monofunctional transpeptidases (3, 4). Since cross-linking of PG is essential in almost all bacteria, inhibiting PBP transpeptidase activity is typically lethal to bacterial cells. Consequently, beta-lactam antibiotics such as penicillin, which inhibit PBPs by covalently bonding to the catalytic serine residue in their transpeptidase domain, are some of the most successful and widely used antibiotics (5). As such, identifying factors that confer resistance to beta-lactam antibiotics and determining their mechanism of action has been an area of significant interest.

**Fig 1:**
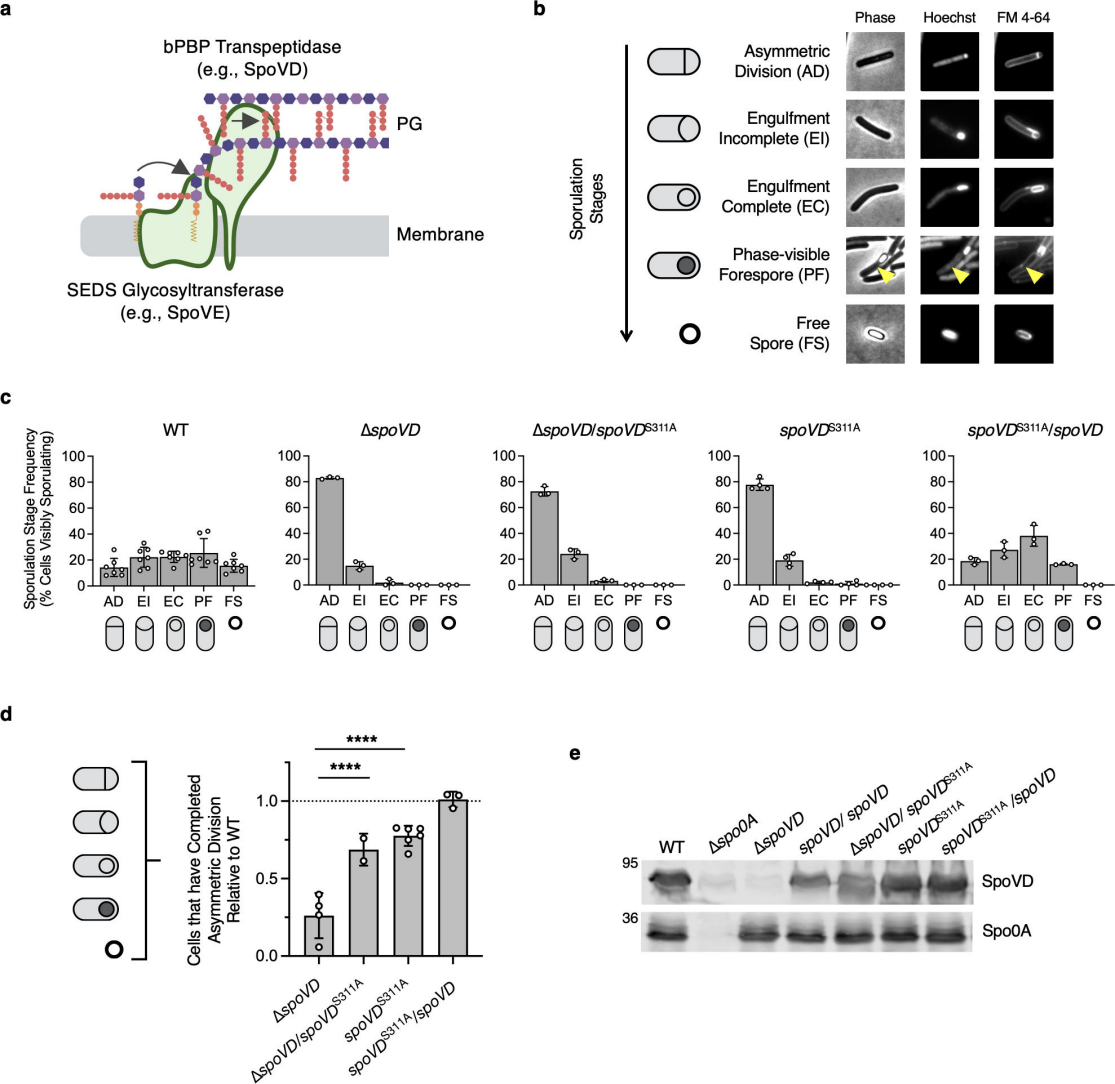
SpoVD catalytic activity is partially dispensable for its function during asymmetric division. (**a**) Schematic of a SEDS-bPBP peptidoglycan synthase complex. The SEDS glycosyltransferase polymerizes nascent glycan strands from lipid-linked PG precursors in the cytoplasmic membrane, while the class B penicillin-binding protein (bPBP) cross-links the stem peptides between the growing strands. (**b**) Cytological profile of individual cells representing each of the five morphological stages of sporulation, as indicated. Representative phase-contrast and fluorescence micrographs show wild-type (WT) cells sampled from sporulation-inducing 70:30 plates after 18 h of growth. The nucleoid was stained using Hoechst, and the cell membrane was stained using FM4-64. Cells undergoing asymmetric division (AD) have a flat polar septum; cells undergoing engulfment (EI) have a curved polar septum; cells that have completed engulfment (EC) are indicated by bright-membrane staining around a fully engulfed forespore; phase-visible forespores (PFs) indicate forespores completing maturation visible as phase-dark or phase-bright forespores (yellow arrowheads) associated with the mother cell; mature free spores (FSs) are observable as independent phase-bright particles. (**c and d**) Quantification of the cytological profiling of cells sampled from sporulation-inducing plates after 20–22 h of growth. White circles indicate data from each replicate; bars indicate the average means; and error bars indicate standard deviation. More than 1,000 total cells and over 100 visibly sporulating cells were analyzed per sample from a minimum of three biological replicates, except for ∆*spoVD*/*spoVD^S311A^*, for which two biological replicates were conducted. For representative micrographs, see Fig. S2. (**c**) Distribution of visibly sporulating cells among the indicated stages of sporulation. (**d**) Proportion of cells that complete and progress beyond asymmetric division, i.e., all visibly sporulating cells, as a percentage of the total cells profiled. Note that the data are normalized to WT (dotted line). ****P < 0.0001. Statistical significance was determined using one-way ANOVA and Tukey’s test. (**e**) Western blot analyses of SpoVD levels in the indicated strains 14 h after growth on sporulation-inducing plates. The anti-Spo0A antibody was used as a proxy for measuring sporulation induction.

One mechanism through which bacteria achieve resistance against beta-lactam antibiotics is by inducing the production of PBPs with lower binding affinities for specific beta-lactams. Most bacteria encode multiple PBPs that are specialized for specific cellular processes; some PBPs are essential, while others can be functionally redundant (3, 4, 6). Essential PBPs typically function as core components of highly conserved multiprotein complexes that drive cell wall synthesis during growth and division. The divisome is the essential complex that drives septal PG synthesis during cell division through the activities of a bPBP transpeptidase that is partnered with a cognate glycosyltransferase of the shape, elongation, division, and sporulation (SEDS) protein family (2, 7, 8). The elongasome is a multiprotein complex that drives cell elongation in rod-shaped bacteria through the action of a distinct SEDS-bPBP pair (2, 9–11). Notably, these SEDS-bPBP pairs are highly specific because the bPBP acts as a selective allosteric activator of its cognate SEDS family glycosyltransferase (11, 12) (Fig. 1a).

Endospore-forming bacteria typically encode an additional SEDS-bPBP complex that drives the morphological changes required for spore formation (13–16). Sporulation begins with the formation of a polar division septum close to one cell pole in a process called asymmetric division. In *Bacillus subtilis*, asymmetric division is driven by the same SEDS-bPBP pair that mediates cell division during vegetative growth (17, 18). In contrast, we recently showed that the spore-forming pathogen *Clostridioides difficile* lacks a canonical division-associated SEDS-bPBP pair for driving septal PG synthesis and instead uses an aPBP as the major PG synthase during vegetative cell division (15). In further contrast with *B. subtilis*, the sporulation-specific SEDS-bPBP pair SpoVE-SpoVD is an important driver of septal PG synthesis during asymmetric division in *C. difficile*. Although the role of SpoVE-SpoVD function during asymmetric division may be restricted to *C. difficile* and other clostridial organisms, genes encoding SpoVE and SpoVD are found in almost all spore formers (13, 14). In both *B. subtilis* and *C. difficile*, SpoVE and SpoVD are essential for synthesizing the spore cortex, a thick layer of modified PG that surrounds and protects the spore core (15, 19–23). Thus, SpoVE and SpoVD are important for synthesizing PG during two distinct stages of spore formation in *C. difficile*.

While a previous study showed that SpoVD’s catalytic activity is essential for spore formation in *C. difficile* (22), in this study, we show that the catalytic activity of SpoVD is not strictly required for mediating asymmetric division despite being essential for synthesizing the cortex layer. Prior analyses of a catalytic mutant of the essential divisome-associated bPBP in *B. subtilis*, PBP2b*_Bs_*, suggest a possible mechanism for explaining this observation. In *B. subtilis*, the catalytic activity of PBP2b*_Bs_* is dispensable because a second bPBP, PBP3*_Bs_*, can supply the transpeptidase activity during septal PG synthesis (24). However, PBP3*_Bs_* cannot complement all the roles fulfilled by the catalytically inactive PBP2b*_Bs_* because the gene encoding PBP2b*_Bs_* is essential, presumably because the PBP2b*_Bs_* protein is required to allosterically activate the glycosyltransferase activity of its SEDS binding partner, FtsW. PBP3*_Bs_* is also important for resistance against certain beta-lactams, since it has lower affinities for them compared to PBP2b*_Bs_* (24). While these observations highlight the importance of catalytic redundancies between PBPs, the molecular mechanisms behind this phenomenon remain unclear.

By examining the mechanism by which *C. difficile* completes asymmetric division in the absence of SpoVD catalytic activity, we demonstrate that the ability of a SpoVD catalytic mutant to support septal PG synthesis during asymmetric division requires the presence of its SEDS partner SpoVE as well as an additional sporulation-induced bPBP, PBP3. We show that PBP3 localizes to the polar septum and can interact, directly or indirectly, with SpoVD, indicating that PBP3 is a component of the PG-synthesizing complex facilitating asymmetric division. Surprisingly, the catalytic activity of PBP3 is dispensable for this compensatory function of PBP3 in promoting asymmetric division in a SpoVD catalytic mutant strain, in contrast with analyses of functional redundancy in *B. subtilis* discussed above (24). Although the precise mechanism by which a PBP3 catalytic mutant enhances asymmetric division in a *C. difficile* SpoVD catalytic mutant strain remains unclear, our analyses reveal that diverse mechanisms enable functional redundancy among PBPs.

## RESULTS

### *C. difficile* SpoVD can facilitate asymmetric division independent of its catalytic activity

Given that the catalytic activity of the division-specific bPBP in *B. subtilis* is not essential for its role in cell division (24), we speculated that the catalytic activity of SpoVD might be similarly dispensable during asymmetric division in *C. difficile*. SpoVD retains the SXXK active-site motif that is highly conserved among bPBPs. To abolish the catalytic activity of SpoVD, we engineered an alanine substitution at the nucleophilic serine residue (SpoVD^S311A^). Consistent with a prior study (22), *C. difficile* strains with a catalytically inactive SpoVD failed to produce heat-resistant spores (Fig. S1). Importantly, this phenotype was observed when *spoVD*^S311A^ was expressed from an ectopic chromosomal locus (∆*spoVD*/*spoVD*^S311A^) or the native locus (*spoVD*^S311A^) (Fig. S1).

To further define the role of SpoVD’s catalytic activity during spore formation, we evaluated the ability of SpoVD catalytic mutant strains to progress through the different morphological stages of sporulation by cytologically profiling sporulating cells (Fig. 1b; Fig. S2) (25, 26). Similar to the *spoVD* deletion strain, *spoVD* catalytic mutant strains failed to produce phase-visible spores, indicating that they have defects in cortex synthesis (Fig. 1c). Furthermore, the distribution of sporulating cells among different stages was similar between the strains, with most cells being stalled at the asymmetric division stage and relatively few cells completing engulfment (Fig. 1c). To specifically quantify the effects on asymmetric division, we calculated the proportion of cells that showed morphological signs of sporulation, i.e., cells that progressed beyond asymmetric division, as a percentage of all cells. These analyses revealed that, relative to wild-type (WT), ∆*spoVD*/*spoVD*^S311A^ and *spoVD*^S311A^ cells complete asymmetric division at significantly higher frequencies compared to ∆*spoVD* cells, with ∆*spoVD* cells completing asymmetric division at ~25% of the levels of WT and ∆*spoVD*/*spoVD*^S311A^ and *spoVD*^S311A^ cells completing asymmetric division at ~70-80% of the levels of WT (Fig. 1d). Since we previously showed that the decreased frequency of visibly sporulating ∆*spoVD* cells reflects a defect in asymmetric division rather than sporulation initiation (15), these results suggest that SpoVD function during asymmetric division is only partially dependent on its catalytic activity. Importantly, the levels of SpoVD present in these strains upon sporulation induction were found to be similar to WT by Western blot analysis (Fig. 1e).

**Fig 2:**
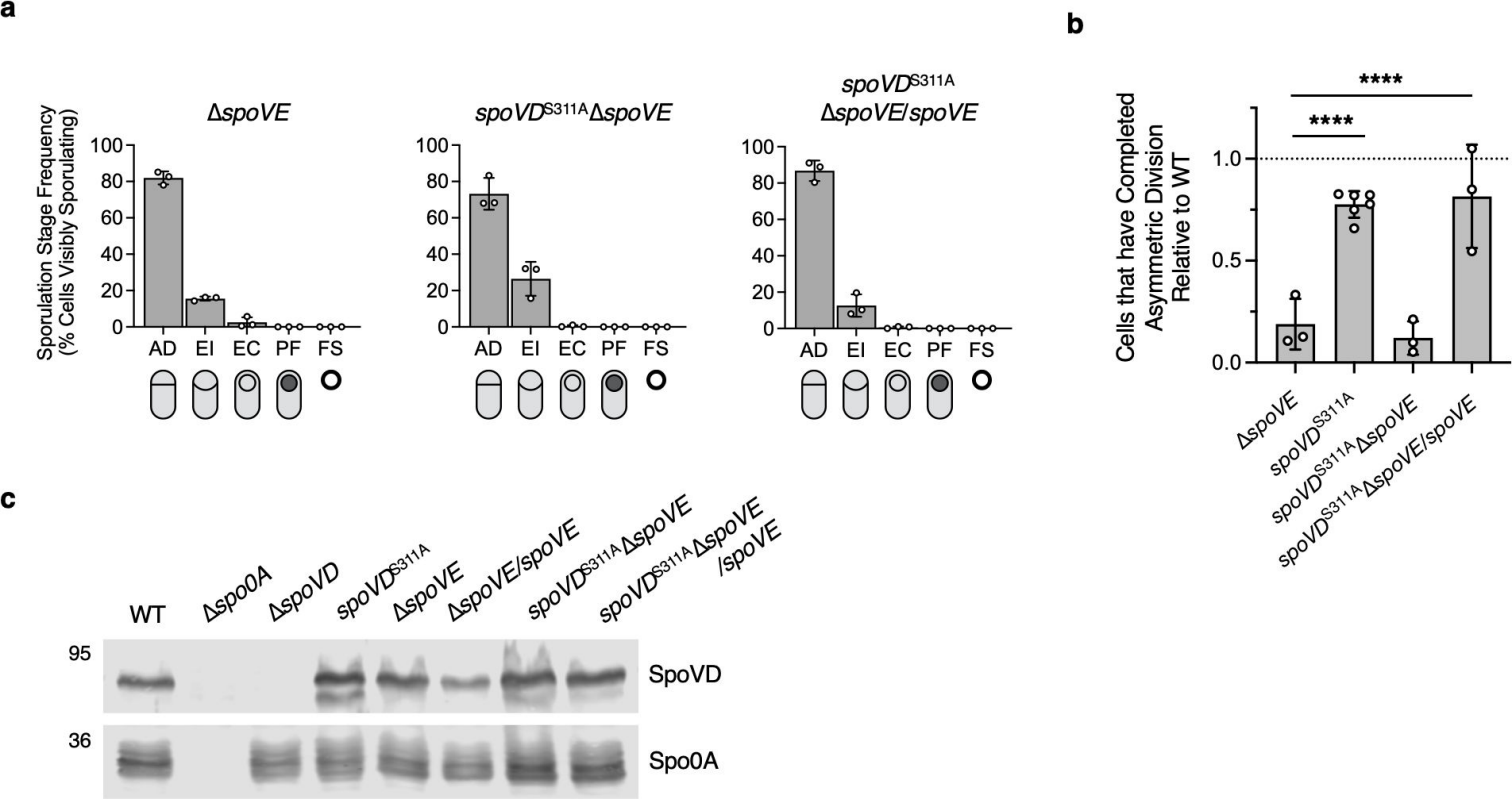
Catalytically inactive SpoVD requires its SEDS partner, SpoVE, to facilitate asymmetric division. (**a and b**) Quantification of the cytological profiling of cells sampled from sporulation-inducing plates after 20–22 h of growth. White circles indicate data from each replicate; bars indicate average means; and error bars indicate standard deviation. More than 1,000 total cells and over 100 visibly sporulating cells were analyzed per sample from a minimum of three biological replicates. (**a**) Distribution of visibly sporulating cells among the indicated stages of sporulation. See Fig. 1 for the distribution of WT cells. (**b**) Proportion of cells that complete and progress beyond asymmetric division, i.e., all visibly sporulating cells, as a percentage of the total cells profiled. Note that the data are normalized to WT (dotted line) and that the *spoVD^S311A^* data were derived from Fig. 1. ****P < 0.0001. Statistical significance was determined using one-way ANOVA and Tukey’s test. (**c**) Western blot analyses of SpoVD levels in the indicated strains 14 h after growth on sporulation-inducing plates. The anti-Spo0A antibody was used as a proxy for measuring sporulation induction.

While we were able to complement the asymmetric division defects of the *spoVD*^S311A^ catalytic mutant strain by expressing a wild-type copy of *spoVD* from an ectopic locus (Fig. 1c and d), this strain was unable to form mature phase-bright spores (Fig. S1 and Fig. 1c). This suggests that the catalytically inactive SpoVD has a dominant-negative phenotype specifically for the defect in cortex synthesis for unknown reasons.

### Catalytically inactive SpoVD requires its SEDS partner, SpoVE, to facilitate asymmetric division

Since previous studies suggest that bPBPs are needed to allosterically activate the glycosyltransferase activity of their cognate SEDS family glycosyltransferases (8, 11, 12), we tested whether the ability of SpoVD^S311A^ to support asymmetric division requires its SEDS partner SpoVE. To this end, we created a *spoVD*^S311A^∆*spoVE* strain by introducing the catalytic mutant variant of *spoVD* into the native locus of a ∆*spoVD*∆*spoVE* strain. As expected, this strain failed to form heat-resistant spores (Fig. S1). Cytological profiling of sporulating cells revealed that *spoVD*^S311A^∆*spoVE* cells complete and progress beyond asymmetric division at a significantly lower frequency (~20%) than WT or *spoVD*^S311A^ cells (100% and ~75%, respectively). Thus, the phenotype of *spoVD*^S311A^∆*spoVE* cells is similar to that of ∆*spoVD* and ∆*spoVE* cells (Fig. 1c and d, 2a and b). Since SpoVD levels are unaffected by loss of SpoVE (Fig. 2c), consistent with our prior results (15), we conclude that the function of SpoVD^S311A^ during asymmetric division requires SpoVE.

### An additional non-essential bPBP, PBP3, is involved in sporulation

**Fig 3:**
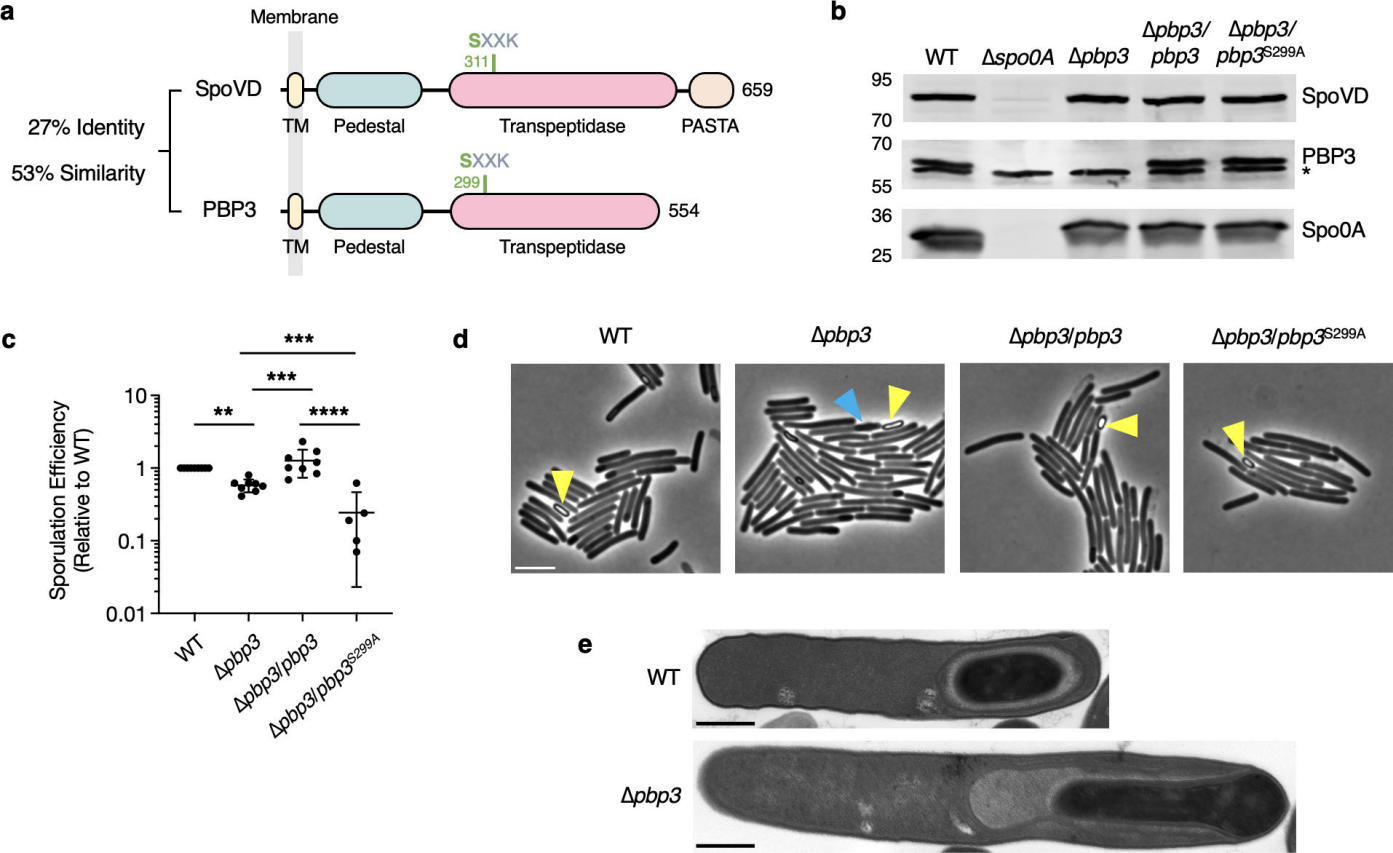
PBP3 is a non-essential bPBP that is involved in spore formation. (**a**) Protein schematic comparing SpoVD and PBP3. Functional domains and catalytic sites were predicted using HMMER (http://hmmer.org/) and revised to reflect the identification of the pedestal domain which interacts with SEDS proteins (11). TM: transmembrane domain; PASTA: PBP and serine/threonine kinase-associated domain. The catalytic serine residues are shown in green. (**b**) Western blot showing the levels of SpoVD, PBP3, and Spo0A in cells sampled from sporulation-inducing plates after ~14 h of growth. SpoVD and PBP3 are not detected in the ∆*spo0A* strain, which cannot initiate sporulation. * indicates a non-specific band detected by the anti-PBP3 antibody. (**c**) Efficiency of heat-resistant spore formation (sporulation efficiency) of the *pbp3* mutant and complemented strains relative to WT. Means with standard deviations are indicated. Cells were collected from sporulation-inducing 70:30 plates ~20 to 22 h after inoculation. Data are from a minimum of five biological replicates. **P < 0.01, ***p < 0.001, ****P < 0.0001. Statistical significance was determined using one-way ANOVA and Tukey’s test. (**d**) Representative phase-contrast micrographs of WT, *pbp3* mutant, and complemented cells collected from sporulation-inducing 70:30 plates after ~20 h of growth. Examples of phase-bright spores are indicated by yellow arrowheads. The blue arrowhead highlights an elongated forespore in the ∆*pbp3* mutant. Scale bar, 5 µm. (**e**) Representative transmission electron microscopy (TEM) images of WT and ∆*pbp3* strains. The forespore of ∆*pbp3* cells was frequently observed to form an elongated shape in the TEM samples. Scale bar, 500 nm.

Next, we wondered if *C. difficile* encodes an additional sporulation-specific bPBP that can cross-link septal PG synthesis in the absence of SpoVD catalytic activity in a manner similar to the functional redundancy observed between division-specific bPBPs in *B. subtilis* (24). Most *C. difficile* strains encode three bPBPs (27, 28): the sole essential bPBP, PBP2, primarily functions during cell elongation, while the two other bPBPs, SpoVD and PBP3, are dispensable for vegetative growth (15). PBP3 is a close homolog of SpoVD containing a similar domain composition apart from the C-terminal PBP and serine/threonine kinase-associated (PASTA) domain carried by SpoVD (Fig. 3a). Both PBP3 and SpoVD have an N-terminal transmembrane domain followed by pedestal and transpeptidase domains.

We considered PBP3 to be a likely candidate for providing functional redundancy to SpoVD during asymmetric division for several reasons. First, previous studies suggest that, similar to *spoVD* expression, the expression of *pbp3* (locus *cd630_12290* in the strain used in this study) is upregulated at the onset of sporulation (29, 30). Second, we previously showed that individual deletions of *spoVD* or *pbp3* do not affect the growth rate of vegetative cells, suggesting that SpoVD and PBP3 play sporulation-specific roles (15). This agrees with our Western blot analysis showing that SpoVD and PBP3 are produced under sporulation-inducing conditions that are dependent on the presence of the master transcriptional regulator of sporulation, Spo0A (Fig. 3b). Taken together with prior transcriptomic studies (29, 30), which similarly show Spo0A-dependent induction of *pbp3* and *spoVD* transcription, these observations demonstrate that both proteins are produced prior to asymmetric division.

Further characterization of the ∆*pbp3* strain revealed that it forms heat-resistant spores ~2-fold less efficiently than WT, a modest defect that can be complemented by expressing *pbp3* from an ectopic chromosomal locus (Fig. 3c). This result is consistent with a prior transposon mutagenesis screen, which identified *pbp3* as being important for sporulation (31). Phase-contrast microscopy of sporulating cells revealed that the ∆*pbp3* mutant forms phase-bright forespores and free spores (Fig. 3d; Fig. S1), indicating that it can assemble the spore cortex, unlike a ∆*spoVD* mutant. Consistent with this conclusion, transmission electron microscopy (TEM) analyses detected the cortex layer in ∆*pbp3* forespores (Fig. 3e). Interestingly, these analyses also revealed that the ∆*pbp3* mutant frequently makes forespores that are elongated and slightly misshapen, indicating that PBP3 affects spore formation downstream of asymmetric division (Fig. 3e). The slight morphological defects may explain the reduction in heat-resistant spore formation relative to WT cells. Notably, while complementation of the ∆*pbp3* mutant with WT *pbp3* restored sporulation efficiency to WT levels, complementation with *pbp3*^S299A^, which encodes a catalytic mutant of PBP3, failed to correct the decrease in heat-resistant spore formation observed in the parental ∆*pbp3* strain. Thus, the catalytic activity of PBP3 is important for proper spore formation (Fig. 3d). Taken together, our results indicate that PBP3 is a sporulation-induced factor that is involved in, but not essential for, the formation of mature spores in *C. difficile*.

### PBP3 promotes asymmetric division in the absence of SpoVD catalytic activity

**Fig 4:**
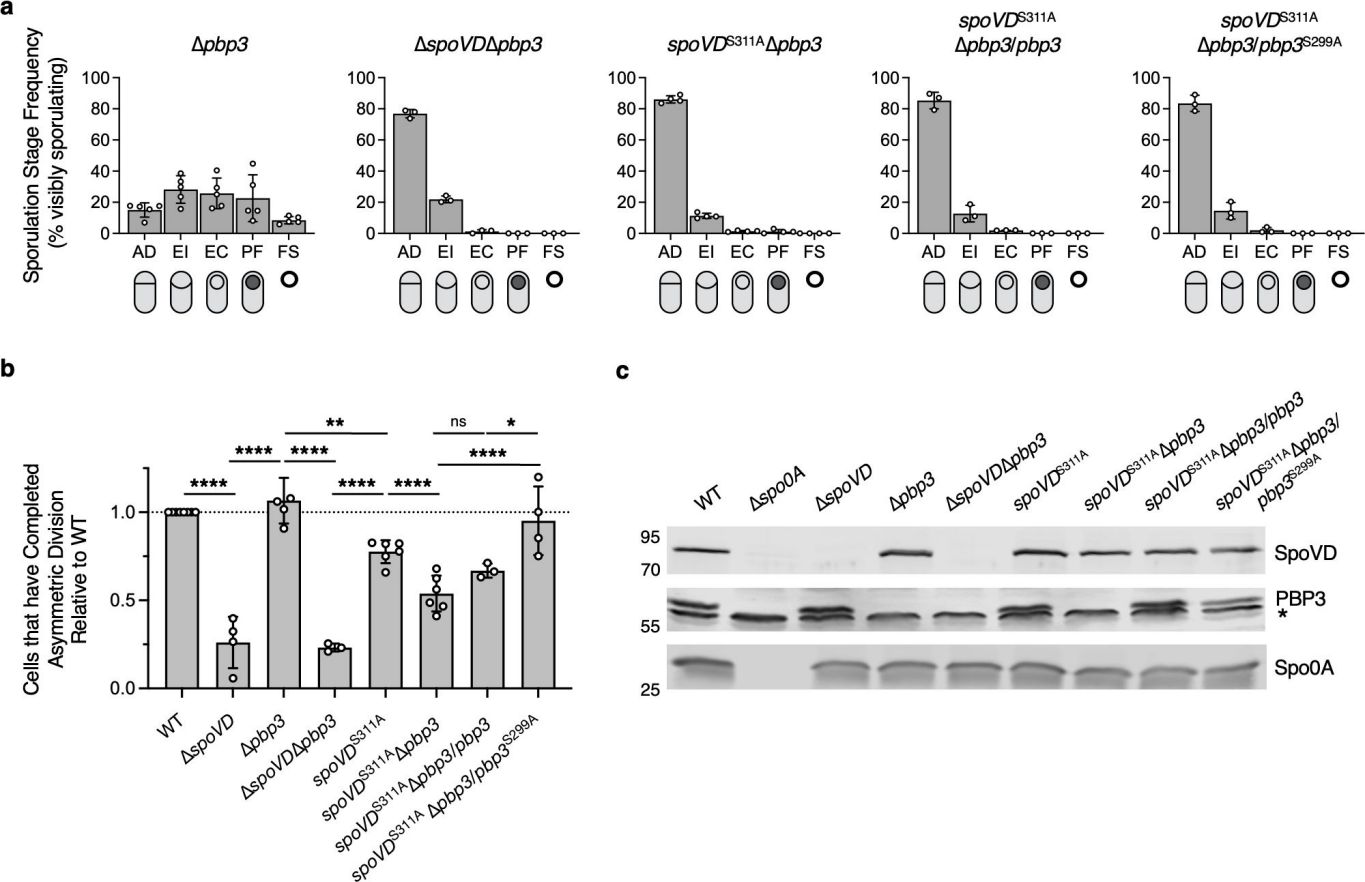
PBP3 partially compensates for the loss of SpoVD catalytic activity during asymmetric division. (**a, b**) Quantification of the cytological profiling of cells sampled from sporulation-inducing plates after 20–22 h of growth. White circles indicate data from each replicate, bars indicate average means, and error bars indicate standard deviation. More than 1,000 total cells and over 100 visibly sporulating cells were analyzed per sample. Data are from a minimum of three independent experiments. For representative micrographs, see Fig. S3. (**a**) Distribution of visibly sporulating cells among the indicated stages of sporulation. See Fig. 1b for the distribution of WT cells. (**b**) Proportion of cells that complete and progress beyond asymmetric division, i.e., all visibly sporulating cells, as a percentage of the total cells profiled. Note that the data are normalized to WT (dotted line) and that the *spoVD^S311A^* data were derived from Fig. 1. ns, not significant. *P < 0.05, **P < 0.01, ****P < 0.0001. Statistical significance was determined using one-way ANOVA and Tukey’s test. (**c**) Western blot showing the levels of SpoVD, PBP3, and Spo0A in cells sampled from sporulation-inducing plates after ~14 h of growth. SpoVD and PBP3 are not detected in the ∆*spo0A* strain, which cannot initiate sporulation. *Indicates a non-specific band detected by the anti-PBP3 antibody.

To test if PBP3 provides redundancy to SpoVD catalytic activity during asymmetric division, we analyzed the ability of ∆*pbp3* cells to complete asymmetric division in the context of WT and catalytically inactive SpoVD variants. The distribution of sporulating ∆*pbp3* cells among the different sporulation stages was similar to WT (Fig. 4a), and cells lacking PBP3 completed asymmetric division at WT levels in the presence of WT SpoVD (Fig. 4b). However, in the *spoVD*^S311A^ background, cells lacking PBP3 completed asymmetric division at a lower rate when compared to WT or *spoVD*^S311A^ cells (Fig. 4b). The proportion of *spoVD*^S311A^∆*pbp3* cells that complete asymmetric division was nevertheless ~2-fold higher compared to cells lacking SpoVD altogether (∆*spoVD*) (53% vs 24%, Fig. 4b and 1d), suggesting that PBP3 partially accounts for the ability of the catalytically dead SpoVD to support asymmetric division. Complementation with WT *pbp3* restored PBP3 protein levels to WT amounts, as measured by Western blot (Fig. 4c), and partially restored the proportion of cells that complete asymmetric division closer to that seen in the *spoVD*^S311A^ mutant, although this small increase in sporulating cells did not reach statistical significance (Fig. 4b). To establish if PBP3’s ability to promote asymmetric division in the *spoVD*^S311A^ background requires the transpeptidase activity of PBP3, we introduced *pbp3*^S299A^ in an ectopic chromosomal locus of a *spoVD*^S311A^ ∆*pbp3* strain. Unexpectedly, cytological profiling of the *spoVD*^S311A^ ∆*pbp3*/*pbp3*^S299A^ strain revealed that these cells complete asymmetric division at levels similar to WT (Fig. 4). While it is unclear why the *spoVD*^S311A^∆*pbp3*/*pbp3*^S299A^ completes asymmetric division at a higher frequency than the *spoVD*^S311A^∆*pbp3*/*pbp3* strain, our data nevertheless suggest that PBP3 enhances asymmetric division in the absence of SpoVD catalytic activity during asymmetric division through a mechanism that is independent of the transpeptidase activity of PBP3.

### PBP3 interacts with components of the polar divisome

**Fig 5:**
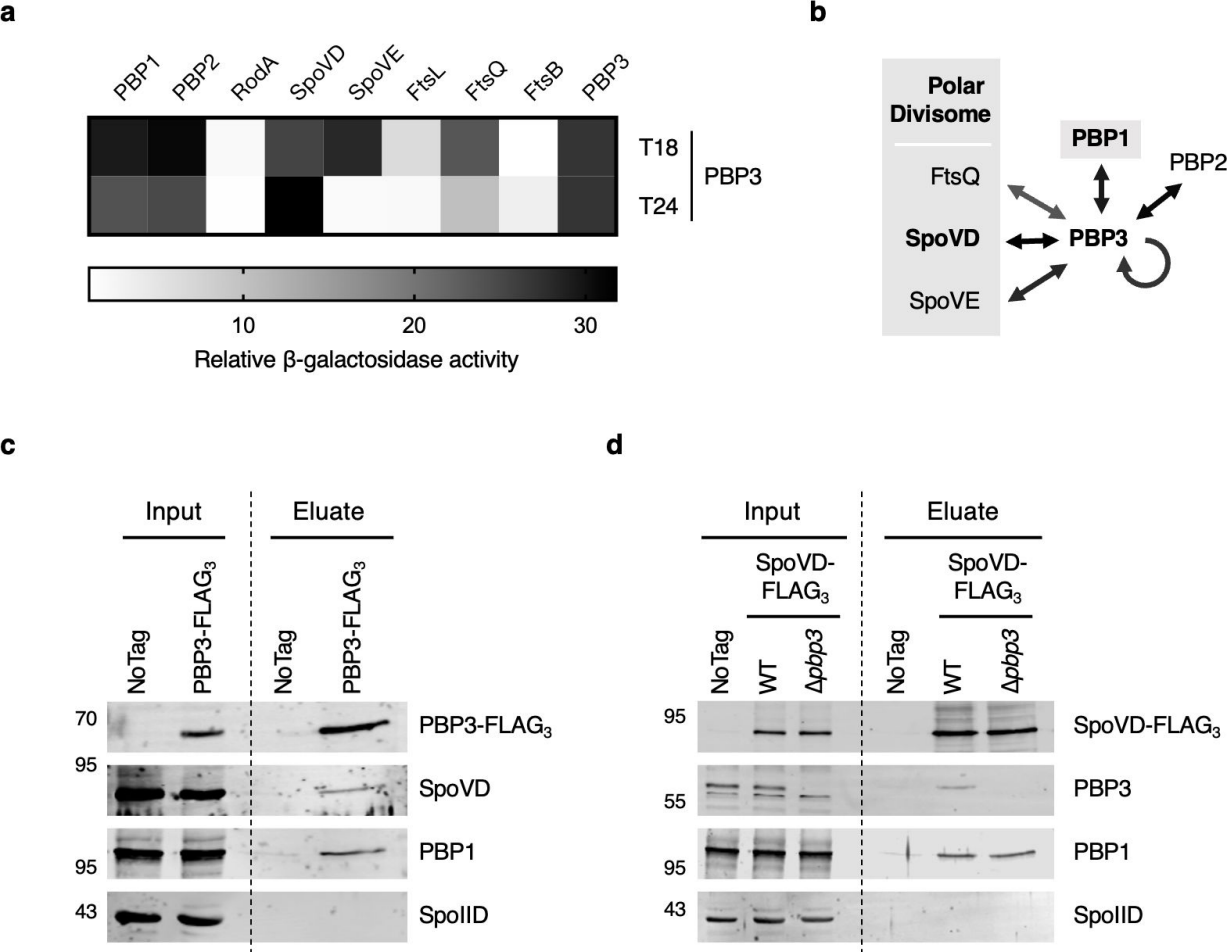
PBP3 interacts with components of the polar divisome. (**a**) Bacterial two-hybrid analysis of interactions between PBP3 and other PG synthases or components of the polar divisome. The β-galactosidase activity was normalized to the negative control. N-terminal T18 or T24 fusion to PBP3 was paired with reciprocal N-terminal fusions to the indicated proteins. Data are from three technical replicates. (**b**) The schematic shows interactions detected in the bacterial two-hybrid analyses. Components of the predicted polar divisome are indicated. PBP1 may also be a part of the polar divisome based on co-immunoprecipitation analyses using SpoVD-FLAG_3_ as bait. (**c and d**) Co-immunoprecipitations performed on cells sampled from sporulation-inducing plates after 12 h of growth. (**c**) PBP3-FLAG_3_ was used as bait in the ∆*pbp3/pbp3-FLAG_3_* strain background; (**d**) SpoVD-FLAG_3_ was used as bait in the ∆*spoVD*/*spoVD-FLAG_3_* (WT) and ∆*spoVD∆pbp3*/*spoVD-FLAG_3_* (∆*pbp3*) strain backgrounds. ∆*pbp3*/*pbp3* and ∆*spoVD/spoVD* strains were used as negative controls (no tag). The presence of SpoVD, PBP3, and PBP1 in the pull-downs was probed using antibodies against the indicated proteins and Western blotting. The FLAG-tagged proteins were detected using an anti-FLAG antibody. SpoIID was used as a control protein because it is also a PG-associated transmembrane protein localized to the forespore membrane, but it is not predicted to be a part of the polar divisome.

We previously demonstrated that SpoVD functions as part of the polar divisome to synthesize septal PG during asymmetric division and showed that it interacts with many divisome-associated proteins (15). Since PBP3 promotes asymmetric division in the *spoVD*^S311A^ mutant, we hypothesized that PBP3 is similarly recruited to the polar division machinery during asymmetric division. To test whether PBP3 interacts with components of the polar divisome, we conducted bacterial two-hybrid assays to probe pairwise interactions between PBP3 and known components of this complex. In addition to SpoVD and SpoVE, we explored interactions with three additional proteins, FtsL, FtsQ, and FtsB (also known as FtsL, DivIB, and DivIC in some Firmicutes), which form a highly conserved divisome sub-complex that regulates PG synthase activity during cell division in other bacteria (15, 32–36). Our data suggest that PBP3 can interact with various components of the polar divisome, including SpoVD, SpoVE, and FtsQ (Fig. 5a and b). We also probed interactions between PBP3 and all other PBP and SEDS family proteins encoded by *C. difficile*. These analyses suggest that PBP3 interacts with PBP1 and PBP2 (Fig. 5a), both of which primarily function during vegetative growth.

Since membrane proteins have a propensity to generate false-positive results in bacterial adenylate cyclase two-hybrid assays by non-specifically reconstituting the adenylate cyclase enzyme (37), we decided to further validate interactions between PBP3 and SpoVD, based on the genetic interaction we observed (Fig. 4). We also tested whether PBP3 and PBP1 interact, given that we previously detected this interaction during vegetative growth and PBP1 is the primary synthase of septal PG in vegetative cells (15, 38). To analyze the potential interactions between PBP3, SpoVD, and PBP1 during *C. difficile* sporulation, we generated ∆*pbp3* and ∆*spoVD* strains complemented with constructs encoding FLAG-tagged PBP3 and FLAG-tagged SpoVD, respectively, for use in co-immunoprecipitation studies. Cell lysates were prepared from cultures grown on sporulation medium for 12 h, when asymmetric division peaks in sporulating cells (39), after which the FLAG-tagged PBPs were pulled down. Western blot analyses of the pull-downs revealed that SpoVD was specifically enriched in PBP3-FLAG_3_ pull-downs (Fig. 5c), and PBP3 was enriched in SpoVD-FLAG_3_ pull-downs (Fig. 5d). Interestingly, PBP1, which is required for mediating vegetative cell division but also likely contributes to asymmetric division (15), was also specifically enriched in the pull-downs, suggesting that PBP1 can interact with one or both SpoVD and PBP3, either directly or indirectly.

To determine whether the interaction between PBP1 and SpoVD depends on the presence of PBP3, we also conducted the co-immunoprecipitation analyses of SpoVD-FLAG_3_ in a ∆*spoVD*∆*pbp3* strain background. PBP1 was still detected in the SpoVD-FLAG_3_ pull-downs in the absence of PBP3, indicating that the interaction between PBP1 and SpoVD does not depend on PBP3. Importantly, a control membrane-bound sporulation protein, SpoIID (40), which is a PG hydrolase that forms part of the engulfment machinery during sporulation (41), failed to be pulled down by either FLAG-tagged SpoVD or FLAG-tagged PBP3 (Fig. 5d). Overall, our data strongly suggest that PBP3 is recruited to the polar divisome complex through interactions with its components (Fig. 5b).

### PBP1 and PBP3 localize to the polar septum during asymmetric division

**Fig 6:**
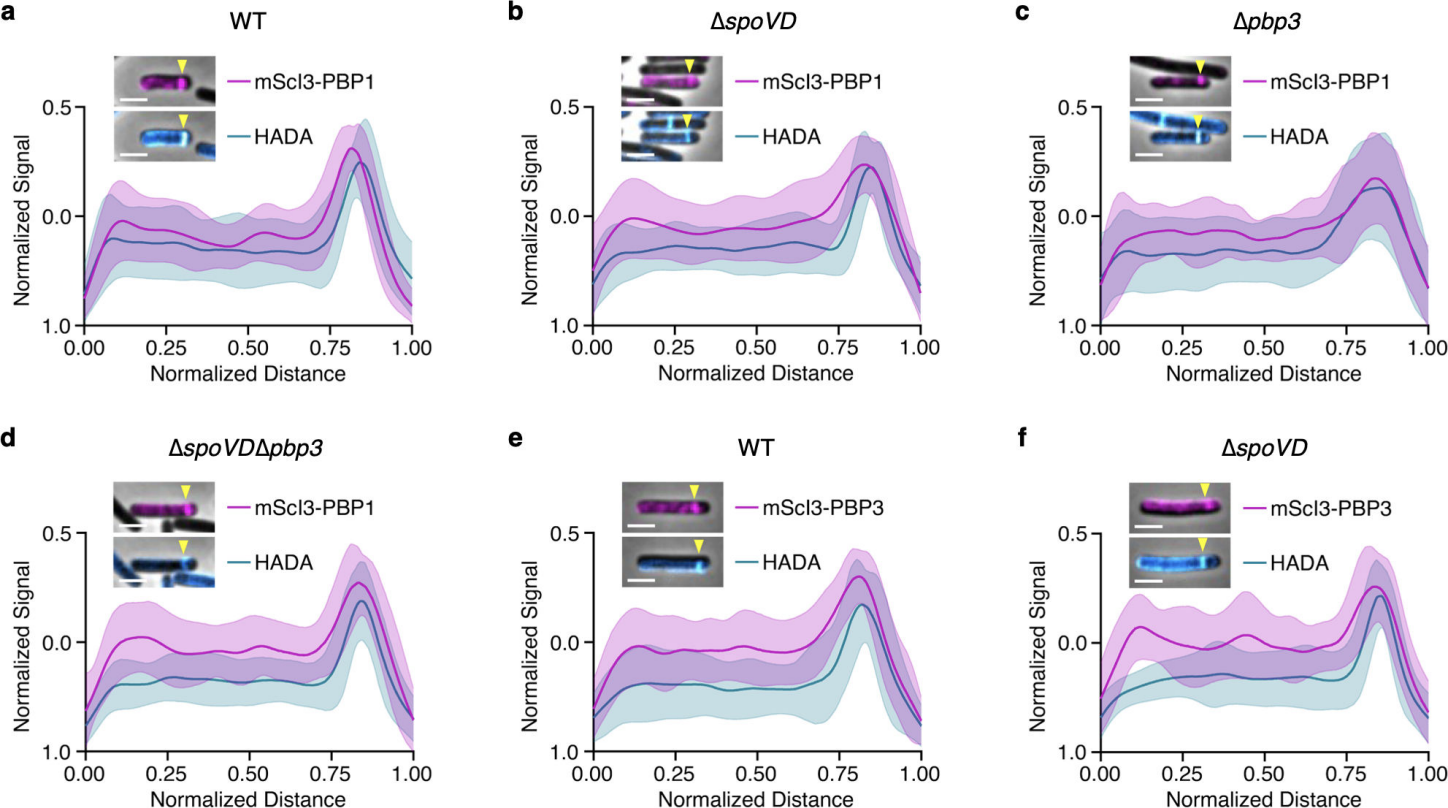
PBP1 and PBP3 localize to the site of asymmetric division. *C. difficile* strains harboring either *mScI3-pbp1* (**a–d**) or *mScI3-pbp3* (**e and f**) expression constructs under an aTc-inducible promoter were grown on sporulating-inducing plates containing 10 ng/mL aTc for 12 h. Cells were then labeled with HADA and fixed for fluorescence microscopy. A representative cell undergoing asymmetric division is shown in the inset for each genotype. The mScI3 and HADA fluorescence along the medial axis of a cell was quantified for 20 individual cells from two independent experiments, and the fluorescent signal was normalized to the maximum fluorescence for each cell before generating aggregate curves. The mean normalized fluorescence ± standard deviation is graphed along the normalized cell distance. The level of induction by aTc on agar medium was variable, likely due to altered diffusion through the bacterial lawn on the agar surface, resulting in some bacteria showing a decreased mScI3 signal (insets in **a–d**). To facilitate comparison between images, the insets in panels **a and d **were adjusted to the same brightness/contrast, and those in panels **e and f** were adjusted to the same brightness/contrast. Yellow arrowheads point to the site of asymmetric division. Scale bars, 2 µm. Images are representative of at least two independent experiments.

Since our protein-protein interaction analyses strongly suggest that PBP3 and PBP1 are constituents of the polar divisome, we next assessed whether these proteins localize to the polar septum. We integrated a construct encoding an mScarlet-I3 (mScI3)-PBP1 fusion under control of the anhydrotetracycline (aTc)-inducible P*_tet_* promoter into an ectopic chromosomal locus (38), generating a merodiploid strain. Upon induction with 10 ng/mL aTc on sporulation-inducing medium, we found that mScI3-PBP1 was enriched at the polar septum during asymmetric division (Fig. 6a). The septal PG was visualized using the fluorescent D-amino acid analog HADA, which is incorporated into sites of PG synthesis and/or remodeling. To determine whether PBP1 localization to the polar septum was dependent on PBP3 and/or SpoVD, we introduced the *mScI3-pbp1* construct into the ∆*pbp3*, ∆*spoVD*, and ∆*spoVD*∆*pbp3* strains. Localization of the mScI3-PBP1 in these mutant backgrounds revealed that PBP1 can localize to the polar septum independent of both PBP3 and SpoVD (Fig. 6b through d). Given that a small proportion of cells can undergo asymmetric division in ∆*spoVD* and ∆*spoVE* mutants relative to WT (Fig. 1 and 2), we suspect that the SpoVD/SpoVE-independent polar septum synthesis is mediated by PBP1, which is the major synthase of septal PG during vegetative growth (15, 38). Our data showing localization of PBP1 to the polar septum independent of SpoVD further support the possibility that PBP1 contributes to the synthesis of the polar septum during spore formation.

We next analyzed the localization of PBP3 during sporulation. We expressed a construct encoding an N-terminal mScarlet-I3 fusion to PBP3 from the aTc-inducible P*_tet_* promoter from the ectopic *pyrE* locus of WT and a ∆*spoVD* mutant. These analyses revealed a similar localization pattern to that of PBP1, with mScI3-PBP3 being enriched at the polar septum during asymmetric division in WT and ∆*spoVD* cells (Fig. 6e and f). Taken together, the localization and interaction analyses strongly suggest that both PBP1 and PBP3 are part of the polar divisome, and their localization to this site does not require SpoVD.

### Endospore-forming bacteria typically encode multiple additional bPBPs compared to non-sporulating bacteria

**Fig 7:**
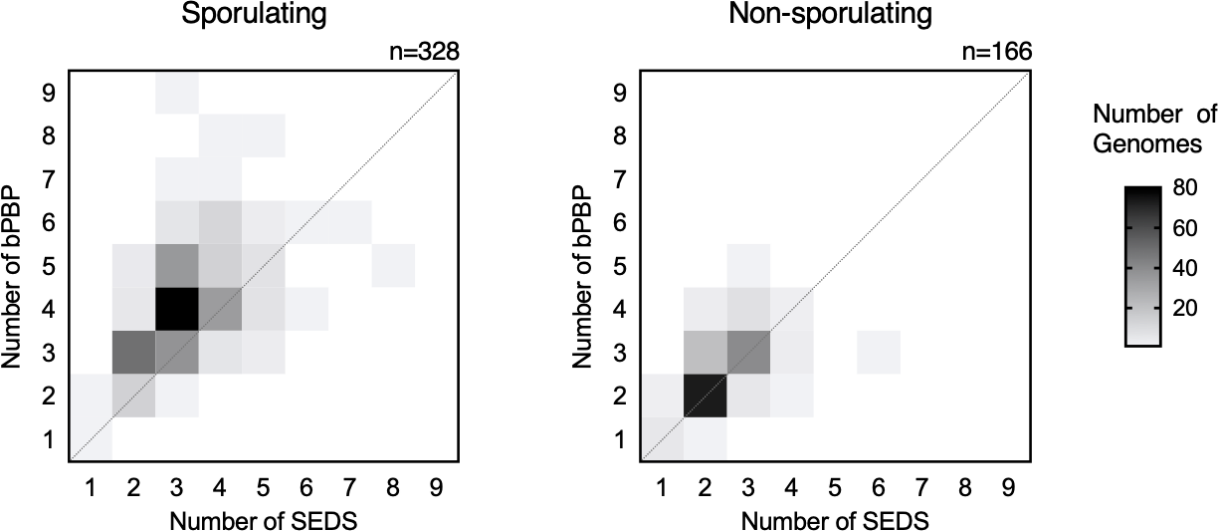
Prevalence of bPBP and SEDS enzymes in Firmicutes. Heatmaps showing the distribution of class B penicillin-binding protein (bPBP) and SEDS protein numbers encoded in the genomes of sporulating (*n* = 328) and non-sporulating (*n* = 166) Firmicutes organisms. Sporulation ability was inferred by the presence of broadly conserved sporulation-specific genes *spo0A* and *spoIIE* in the genome. The data set comprises 494 diverse Firmicutes organisms as reported in reference 15.

Since these analyses revealed functional redundancies between sporulation-induced *C. difficile* bPBPs, we wondered whether this phenomenon might be broadly conserved in endospore-forming bacteria. To this end, we compared the numbers of SEDS and bPBP enzymes encoded in the genomes of non-sporulating and sporulating Firmicutes, the sole bacterial phylum with endospore-forming members. Our analyses revealed that sporulating bacteria typically encode a higher number of bPBPs compared to non-sporulating bacteria (Fig. 7). In sporulating organisms, the number of encoded bPBPs often exceeds the number of encoded SEDS (214/328, 65%), while only a small minority of organisms encode more SEDS proteins than bPBPs (20/328, 6%). Furthermore, a higher percentage of non-sporulating organisms encode equal numbers of SEDS and bPBP genes (80%). These observations are consistent with the prevalence of additional sporulation-specific PG synthases in spore formers and suggest that redundancy in bPBP activity is likely widespread in these organisms.

## DISCUSSION

While most bacteria encode multiple bPBPs that perform specialized roles during specific cellular processes, some bPBPs can play redundant roles under certain growth conditions, including environmental stress (6, 24, 42–45). Our understanding of these compensatory mechanisms and their prevalence, however, remains incomplete. Here, we reveal that the catalytic activity of SpoVD in *C. difficile* is partially dispensable for its function during asymmetric division (Fig. 1). Our data indicate that a sporulation-induced bPBP, PBP3, partially substitutes for the loss of SpoVD’s catalytic activity during this sporulation-specific division mechanism because loss of PBP3 in a *spoVD* catalytic mutant strain (*spoVD^S311A^*) reduces the ability of this mutant to complete asymmetric division (Fig. 1 and 4). Notably, while this finding is analogous to how *B. subtilis* PBP3*_Bs_* partially substitutes for the function of PBP2B*_Bs_* (24) and *Enterococcus faecalis* Pbp4*_Ef_* partially substitutes for the function of PbpB*_Ef_* (45) during vegetative cell division, the catalytic activity of PBP3 in *C. difficile* is dispensable for promoting the ability of a SpoVD catalytic mutant to complete asymmetric division (Fig. 4), in contrast with the *B. subtilis* and *E. faecalis* systems.

While the precise mechanism by which PBP3 modulates SpoVD function during asymmetric division remains unclear, our interaction and localization analyses establish PBP3 as a component of the polar divisome complex (Fig. 5 and 6). These analyses also identify the class A PBP, PBP1, as a constituent of the polar divisome (Fig. 5 and 6) and a likely driver of asymmetric division in the absence of SpoVD or SpoVE. This latter conclusion is based on our finding that PBP1 localizes to the asymmetric division site in WT and ∆*spoVD* cells (Fig. 6), interacts with members of the polar divisome (Fig. 5), and drives vegetative cell division in *C. difficile* (15). However, PBP1 is considerably less efficient than the SpoVE-SpoVD PG synthase complex at completing asymmetric division, given that the ∆*spoVD* and ∆*spoVE* mutants complete this stage at ~25% the frequency of WT (Fig. 1 and 2).

Notably, *C. difficile* PBP1 appears to mediate asymmetric division through a mechanism that does not depend on PBP3, despite our recent finding that PBP3 promotes the activity of PBP1 during vegetative cell division (38). This conclusion is based on our finding that ∆*spoVD* and ∆*spoVD*∆*pbp3* mutants complete asymmetric division at similar frequencies (Fig. 4b) and that PBP1 localizes to the polar septum even in the absence of SpoVD and/or PBP3 (Fig. 6). Future work is required to understand how PBP1 might be coordinated with SpoVD-SpoVE and PBP3 during asymmetric division in *C. difficile*. Intriguingly, the requirement for the activities of both a SEDS-bPBP complex and an aPBP for septal PG synthesis during asymmetric division may be more widely conserved, given that a strain lacking the major aPBP PBP1*_Bs_* in *B. subtilis* is deficient in completing asymmetric division (46).

Our data further show that PBP3 facilitates asymmetric division through a mechanism that depends on SpoVD but is independent of the catalytic activity of PBP3. Yet, the enzymatic activity of PBP3 is nevertheless important for events downstream of asymmetric division because a ∆*pbp3* mutant exhibits a modest but consistent ~2-fold defect in functional spore formation relative to WT, while a PBP3 catalytic mutant exhibits an ~5-fold defect relative to WT (Fig. 3). Although it is unclear how PBP3 promotes functional spore formation downstream of asymmetric division, we frequently observed that sporulating ∆*pbp3* cells produce abnormally long forespores that are often found flush with the cell pole (Fig. 3e and Fig. S3). This morphological defect could be caused by an inability to complete membrane fission (47). It is also unclear why the sporulation defect of the PBP3 catalytic mutant complementation strain (∆*pbp3*/*pbp3^S299A^*) is more severe than that of the parental ∆*pbp3* strain (Fig. 3c). Since PBP3 can directly interact with the SEDS glycosyltransferase SpoVE in the bacterial two-hybrid assay (Fig. 5a), one possibility is that the PBP3 catalytic mutant competes with SpoVD for binding to SpoVE and thus impairs SpoVD function. Such a scenario would be consistent with recent work suggesting that SEDS glycosyltransferases can interact with more than one bPBP. For example, in the facultative pathogen *Salmonella enterica*, different bPBPs exhibit niche specialization during extracellular versus intracellular conditions (42, 43). In particular, two division-specific *S. enterica* bPBPs appear to function independently of the other in their respective niches (42). Thus, either enzyme may be capable of interacting with the division-specific SEDS glycosyltransferase, FtsW (42). Similarly, elongasome function in *B. subtilis* can be supported by two distinct bPBPs, PBP2a*_Bs_* and PBPH*_Bs_* (48, 49), implying that either bPBP can complex with the elongation-specific SEDS protein, RodA, and regulate its function.

In contrast, the functional redundancy reported between the division-associated bPBPs, PBP3*_Bs_* and PBP2b*_Bs_*, requires the presence of the catalytically inactivated PBP2b*_Bs_* protein (24). Similarly, the redundancy observed in *E. faecalis* is thought to require direct interactions between two bPBPs within the larger divisome complex (45). In these examples, specific SEDS-bPBP pairs likely act as cognate pairs, with the bPBP playing a structural role in allosterically activating the glycosyltransferase activity of the SEDS enzyme (8, 11, 12). In this case, while an additional bPBP might be recruited to complex and provide catalytic redundancy, this functionally redundant bPBP is unable to replace the primary bPBP in interacting with and stimulating the activity of its SEDS partner.

Since the ability of *C. difficile* PBP3 to promote asymmetric division depends on the presence of SpoVD (Fig. 4), the redundancy between SpoVD and PBP3 bPBPs appears to be more similar to the catalytic redundancy observed between division-specific bPBPs in *B. subtilis* and *E. faecalis*. Thus, we favor a model in which *C. difficile* SpoVD allosterically activates the glycosyltransferase activity of SpoVE, while PBP3 functions as an auxiliary factor, rather than a replacement for SpoVD, in the polar divisome complex. Given that the catalytic activity of PBP3 is dispensable for this function, other enzyme(s) must be capable of generating the peptide cross-links in the *spoVD^S311A^∆pbp3*/*pbp3^S299A^* mutant. Since the remaining *C. difficile* bPBP, PBP2, is specialized for elongation (15), either PBP1, the sole aPBP in *C. difficile*, or one of the five L,D-transpeptidases encoded by *C. difficile* likely supplies this activity (50).

Another question raised by our study is why the catalytic activity of SpoVD is essential for cortex synthesis but largely dispensable for asymmetric division. One possibility is that cortex synthesis involves SpoVE-SpoVD functioning as part of a larger PG synthetic complex, similar to the divisome and elongasome. Since SpoVE-SpoVD function during this process requires their specific localization to the forespore, other factors are likely involved in their regulation. Notably, one factor that does not appear to be critical for cortex synthesis is PBP3, since ∆*pbp3* sporulating cells can form a cortex layer and purified ∆*pbp3* spores exhibit similar morphologies as WT spores (Fig. S4).

Regardless of the mechanism by which non-essential bPBPs can partially substitute for the function of essential bPBPs involved in cell division, redundancy in bPBP activity is likely widespread among spore-forming bacteria. Indeed, we find that these bacteria typically encode multiple additional bPBPs, with the number of bPBPs often exceeding the number of SEDS glycosyltransferases encoded in the genome (Fig. 7). Defining specialized roles or redundancies between these enzymes requires further study and may have important implications for the ability of these organisms to respond to environmental and antibiotic stress. For example, while most *C. difficile* strains carry five distinct HMW PBPs, a study characterizing clinical strains identified an additional bPBP encoded by *Clostridium difficile* ribotype 017 isolates (27, 28). Some of these isolates are less sensitive to the beta-lactam imipenem and carry mutations in the transpeptidase catalytic sites of the division-associated PBP1 and elongation-associated PBP2. The additional bPBP encoded by these strains, therefore, may additionally contribute to imipenem resistance by providing functional redundancy to these enzymes or SpoVD, recapitulating the scenario observed in MRSA strains. Taken together with our findings, these observations highlight the need to define functional redundancies between PBPs in *C. difficile*.

## MATERIALS AND METHODS

### *C. difficile* strain construction and growth conditions

All *C. difficile* strains are derived from the 630∆*erm* strain. Deletion and complementation strains were constructed in a ∆*pyrE* background strain using *pyrE*-based allele-coupled exchange as previously described (51). All strains used in the study are reported in Table S1. Strains were grown at 37°C under anaerobic conditions using a gas mixture containing 85% N_2_, 5% CO_2_, and 10% H_2_.

### *Escherichia coli* strain constructions

Table S2 lists all plasmids used in the study, with links to plasmid maps containing all prime sequences used for cloning. Plasmids were cloned via Gibson assembly, and cloned plasmids were transformed into *E. coli* (DH5α or XL1-Blue strains). All plasmids were confirmed by sequencing the inserted region. Confirmed plasmids were transformed into the *E. coli* HB101(pRK24) strain for conjugation with *C. difficile* when needed. All *E. coli* HB101 strains used for conjugation are indicated in Table S2.

### Plate-based sporulation assays

For assays requiring sporulating cells, cultures were grown to early stationary phase, back-diluted >25-fold into BHIS, and grown until they reached exponential phase (OD_600_ between 0.35 and 0.75). Exponentially growing cells (120 µL) were spread onto 70:30 (70% SMC media and 30% BHIS media) agar plates (40 mL media per plate). After 18–22 h of growth, sporulating cells were collected into phosphate-buffered saline (PBS), and sporulation levels were visualized by phase-contrast microscopy as previously described (52).

### Heat-resistance assay

Heat-resistant spore formation was measured 20–22 h after sporulation induction on 70:30 agar plates by resuspending sporulating cells in PBS, dividing the sample into two, heat-treating one of the samples at 60°C for ~30 min, and comparing the colony-forming units (CFUs) in the untreated sample to the heat-treated sample (53). Heat-resistance efficiencies represent the average ratio of heat-resistant CFUs to total CFUs for a given strain relative to the average ratio for the wild-type strain.

### Fluorescence and phase-contrast microscopy

Fluorescence microscopy was performed on sporulating cells using Hoechst 33342 (Molecular Probes, 15 µg/mL) and FM4-64 (Invitrogen, 1  µg/mL) to stain the nucleoid and membrane, respectively. All samples for a given experiment were imaged from a single agar pad (1.5% low-melting-point agarose in PBS).

Phase-contrast images in Fig. 3 were obtained using a Zeiss Axioskop upright microscope with a ×100 Plan-NEOFLUAR oil-immersion phase-contrast objective and a Hamamatsu C4742-95 Orca 100 CCD Camera. All other phase-contrast and fluorescence images were acquired using a Leica DMi8 inverted microscope with a ×63 1.4 NA Plan Apochromat oil-immersion phase-contrast objective, a high-precision motorized stage (Pecon), and an incubator (Pecon) set at 37°C. Excitation light was generated by a Lumencor Spectra-X multi-LED light source with integrated excitation filters. An XLED-QP quadruple-band dichroic beam splitter (Leica) was used (transmission: 415, 470, 570, and 660 nm) with an external filter wheel for all fluorescent channels. FM4-464 was excited at 555/28 nm and emitted light was filtered using a 705/72 nm emission filter (Leica). Hoechst and HADA were excited at 395/25 nm, and emitted light was filtered using a 440/40 nm emission filter (Leica); mScarlet-I3 was excited at 555/28  nm, and emitted light was filtered using a 590/50 nm emission filter (Leica). Emitted and transmitted light was detected using a Leica DFC 9000 GTC sCMOS camera. One to two micrometer z-stacks were taken when needed with 0.21 µm z-slices.

Images were acquired and exported using the LASX software without further processing, with the exception of mScI3-PBP1 and mScI3-PBP3 localization images, which were deconvolved using Leica Small Volume Computational Clearing with the following settings: refractive index 1.33, strength 60%, and regularization 0.05. After export, images were processed using Fiji (54) to remove out-of-focus regions, and the best-focused z-planes for all channels were manually selected. Image scaling was adjusted to improve brightness and contrast for display and was applied equally to all images shown in a single panel unless stated otherwise in the figure legend. Visualization of quantified data and any associated statistical tests was performed using Prism 10 (GraphPad Software, San Diego, CA, USA).

### Localization of mScI3-tagged PBP1 and PBP3

*C. difficile* harboring a *mScI3-pbp1* or *mScI3-pbp3* expression cassette under control of the aTc-inducible P*_tet_* promoter integrated downstream of the *pyrE* locus was grown to mid-log and spread on 70:30 agar plates containing 10 ng/mL aTc to induce expression of the constructs during sporulation. The constructs harbor a (GGGGS)_3_ linker between the mScI3 fluorescent protein and the protein of interest. After 12 h, a sample of *C. difficile* was scraped into 500 µL BHIS containing 50 µM HADA (Tocris), allowed to label for 10 min at 37°C, then fixed with a mixture of 100 µL 16% paraformaldehyde and 20 µL 1 M NaPO_4_ buffer (pH 7.4) for 30 min at room temperature and 30 min on ice. After cell fixation, cells were washed three times with 1 mL of PBS and incubated overnight at room temperature in the dark to enable chromophore maturation before fluorescence microscopy imaging, as previously described (55).

### Quantification of mScI3-PBP1/PBP3 and HADA fluorescence along the medial axis

The fluorescence signal of mScI3-PBP1, mScI3-PBP3, and/or HADA was measured along the medial axis of sporulating cells using the line scan tool in Fiji. To combine data from multiple cells into an aggregate curve, the normalized fluorescence was calculated based on the maximum fluorescence along each cell, and the normalized distance was calculated relative to the size of each cell. The curves for each cell were plotted in GraphPad Prism, and a LOWESS curve for fine smoothing was fitted to each cell in order to average data from multiple cells on a single graph. For the aggregate data, the mean normalized fluorescence was plotted along the normalized distance of each data set.

### Transmission electron microscopy

Sporulating cells were collected ~22  h after sporulation induction on 70:30 or SMC agar plates. Cells were fixed and sent for processing for electron microscopy by the University of Vermont Microscopy Center, as previously described (15, 56). All TEM images were captured on a JEOL 1400 Transmission Electron Microscope (Jeol USA, Inc., Peabody, MA, USA) with an AMT XR611 high-resolution 11 megapixel mid-mount CCD camera.

### Western blot analysis

Samples were collected 18–22 h after sporulation induction on 70:30 agar plates and processed for immunoblotting. Sample processing involved multiple freeze-thaws in PBS followed by the addition of EBB buffer (9 M urea, 2 M thiourea, 4% SDS, and 2 mM b-mercaptoethanol), boiling, pelleting, resuspension, and boiling again before loading on a gel. All proteins were resolved using 4%–15% precast polyacrylamide gels (Bio-Rad) and transferred to polyvinylidene difluoride membranes, which were subsequently probed with rabbit anti-SpoVD (15) (both at 1:1,000 dilution), mouse anti-Spo0A (29) (at 1:1,000 dilution), and chicken anti-GDH (at 1:5,000 dilution) polyclonal primary antibodies, and antirabbit (IR800 or IR680), antimouse (IR680), and antichicken (IR800) secondary antibodies (LI-COR Biosciences; 1:20,000 dilution). Blots were imaged using the LiCor Odyssey CLx imaging system. The results shown are representative of multiple experiments.

To detect PBP3 using Western blots, cell lysates were prepared from sporulating cultures after 14 h of growth on sporulation-inducing medium using a bead-beating approach. In particular, cells were pelleted, resuspended in 750 µL of FLAG IP Buffer (150 mM NaCl, 50 mM Tris-HCl, pH 7.5), transferred to screwcap tubes containing MP Biomedicals Lysing Matrix, and frozen at -80°C. Frozen cells were thawed and bead-beaten on an MP Biomedicals FastPrep-24 four times at 5.5 M/second for 1 min, resting on ice for 5 min between rounds of bead-beating. Next, 1× HALT protease inhibitors and dodecyl-β-d-maltoside (DDM) detergent were added to lysed cells to a final concentration of 0.5%, and mixtures were rotated at room temperature for 1 h to solubilize membrane proteins. Lysates were clarified by centrifuging at 10,000 × *g* for 1 min. The soluble lysates were then resolved using SDS-PAGE and analyzed using Western blots.

### Antibody production

Anti-PBP3 and anti-PBP1 antibodies used for Western blots in this study were raised in rabbits by Cocalico Biologicals against PBP3 and PBP1 variants lacking the transmembrane domains. PBP3_Δ1-35_-His_6_ and His_6_-PBP1_Δ1-77_ were produced in BL21(DE3) *E. coli* harboring pET28a-*pbp3*_Δ1-35_-His_6_ and pET28a-His_6_-*pbp1*_Δ1-77_, respectively, and purified by Ni^2+^-affinity purification as previously described (29). Antisera reactivity and specificity for PBP3 and PBP1 were validated by Western blot against *C. difficile* lysate from WT and either a Δ*pbp3* mutant (this work) or a *pbp1* CRISPR-interference knock-down strain (15), respectively.

### Bacterial two-hybrid analyses

Bacterial adenylate cyclase two-hybrid (BACTH) assays were conducted as previously described (57) using *E. coli* BTH101 cells. Briefly, BTH101 cells were transformed with 100 ng of each plasmid and plated on LB agar plates supplemented with 50 µg/mL kanamycin, 100 µg/mL ampicillin, and 0.5 mM isopropyl β-D-thiogalactopyranoside (IPTG). Plates were incubated for 64–68 h at 30°C, and β-galactosidase activity was quantified in Miller units as previously detailed (58). The β-galactosidase activity of cells transformed with the empty pUT18C and pKT25 vectors was used as a negative control for normalization.

### Co-immunoprecipitation (Co-IP) analyses

To immunoprecipitate FLAG-tagged proteins from sporulating *C. difficile*, exponentially growing cultures of *C. difficile* harboring *pbp3-FLAG_3_* or *spoVD-FLAG_3_* expression constructs and appropriate control strains were spread onto 70:30 agar plates. After 12 h of growth on 70:30 plates, sporulating cells were scraped from 3 plates per strain and pooled in 1 mL of PBS. A portion of cells was visualized by microscopy with peptidoglycan labeling to confirm the presence of sporulating cells undergoing asymmetric division in each sample. Cells were cross-linked with 0.25% final concentration of PFA for 15 min at 37°C and quenched with 350 mM glycine for 10 min on ice. Cross-linked cells were then pelleted, resuspended in 750 µL of FLAG IP Buffer (150 mM NaCl, 50 mM Tris-HCl pH 7.5), transferred to screwcap tubes containing MP Biomedicals Lysing Matrix E, and frozen at -80°C. Frozen cells were thawed and bead-beaten on an MP Biomedicals FastPrep-24 four times at 5.5 M/second for 1 min, resting on ice for 5 min between rounds of bead-beating. Next, 1× HALT protease inhibitors and dodecyl-β-d-maltoside (DDM) detergent were added to lysed cells to a final concentration of 0.5%, and mixtures were rotated at room temperature for 1 h to solubilize membrane proteins. Lysates were clarified by centrifuging at 10,000 × *g* for 1 min, and 200 µL of pre-equilibrated Anti-FLAG M2 Magnetic Dynabead resin was added to the clarified lysate. The lysate-resin mixture was rotated at room temperature for 1 h. To remove unbound proteins, the resin was washed three times briefly and once for 5 min with 1 mL FLAG IP Buffer containing 0.5% DDM. This step was repeated a total of two times. Then, the resin was washed three times briefly and once for 5 min with FLAG IP Buffer containing no detergent. Finally, bound proteins were eluted from the resin with 100 µg/mL 3× FLAG peptide, boiled in 1× sample buffer for 10 min to reverse cross-links, and then analyzed by Western blot using rabbit polyclonal antibodies against PBP1 (TF134, this study), PBP3 (TF135, this study), SpoVD (TF124 [15]), and SpoIID (TF121 [40]) or a mouse monoclonal M2 anti-FLAG antibody (Sigma).

## SUPPLEMENTAL MATERIAL

Supplemental figures and tables jb.00503-25-s0001.pdf Figures S1 to S4, and Tables S1 and S2.

ASM does not own the copyrights to Supplemental Material that may be linked to, or accessed through, an article. The authors have granted ASM a non-exclusive, world-wide license to publish the Supplemental Material files. Please contact the corresponding author directly for reuse.
