# Supplementary material for: Penicillin-binding proteins exhibit functional redundancy during asymmetric cell division in Clostridioides difficile
Source: J Bacteriol. 2025 Nov 26;207(12):e00503-25. doi: 10.1128/jb.00503-25 (PMC12713402; doi:10.1128/jb.00503-25)
Supplement: Supplemental figures and tables — Figures S1 to S4, and Tables S1 and S2. [file jb.00503-25-s0001.pdf]

Supplemental Figures and Tables

Penicillin-binding proteins exhibit functional redundancy during asymmetric cell division in *Clostridioides difficile*

Shrestha *et al*.

Figures S1 to S4

Tables S1 and S2

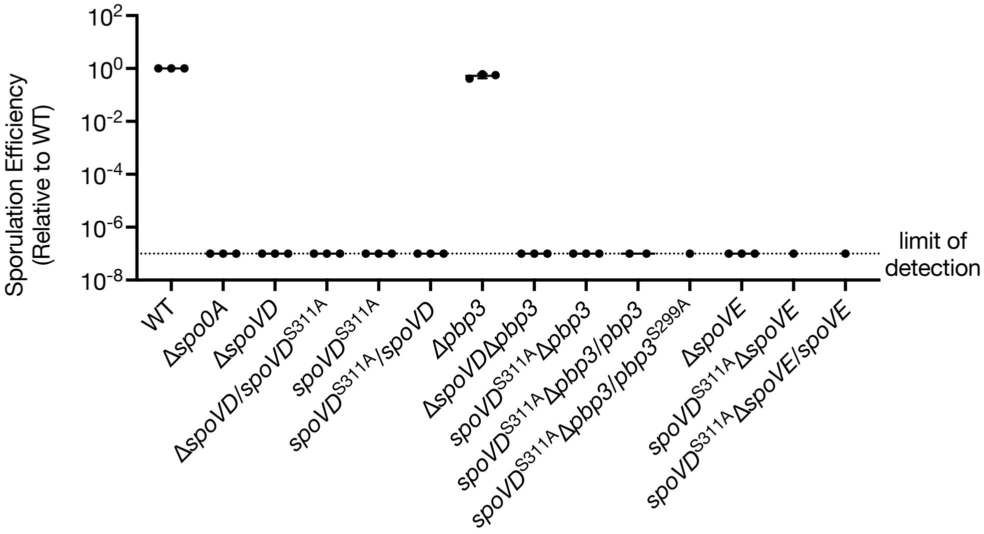

FIG S1 The efficiency of heat-resistant spore formation (sporulation efficiency) of various mutant and complemented strains relative to WT. Means with standard deviation are indicated. Cells were collected from sporulation-inducing 70:30 plates ~20-22 hours after inoculation.

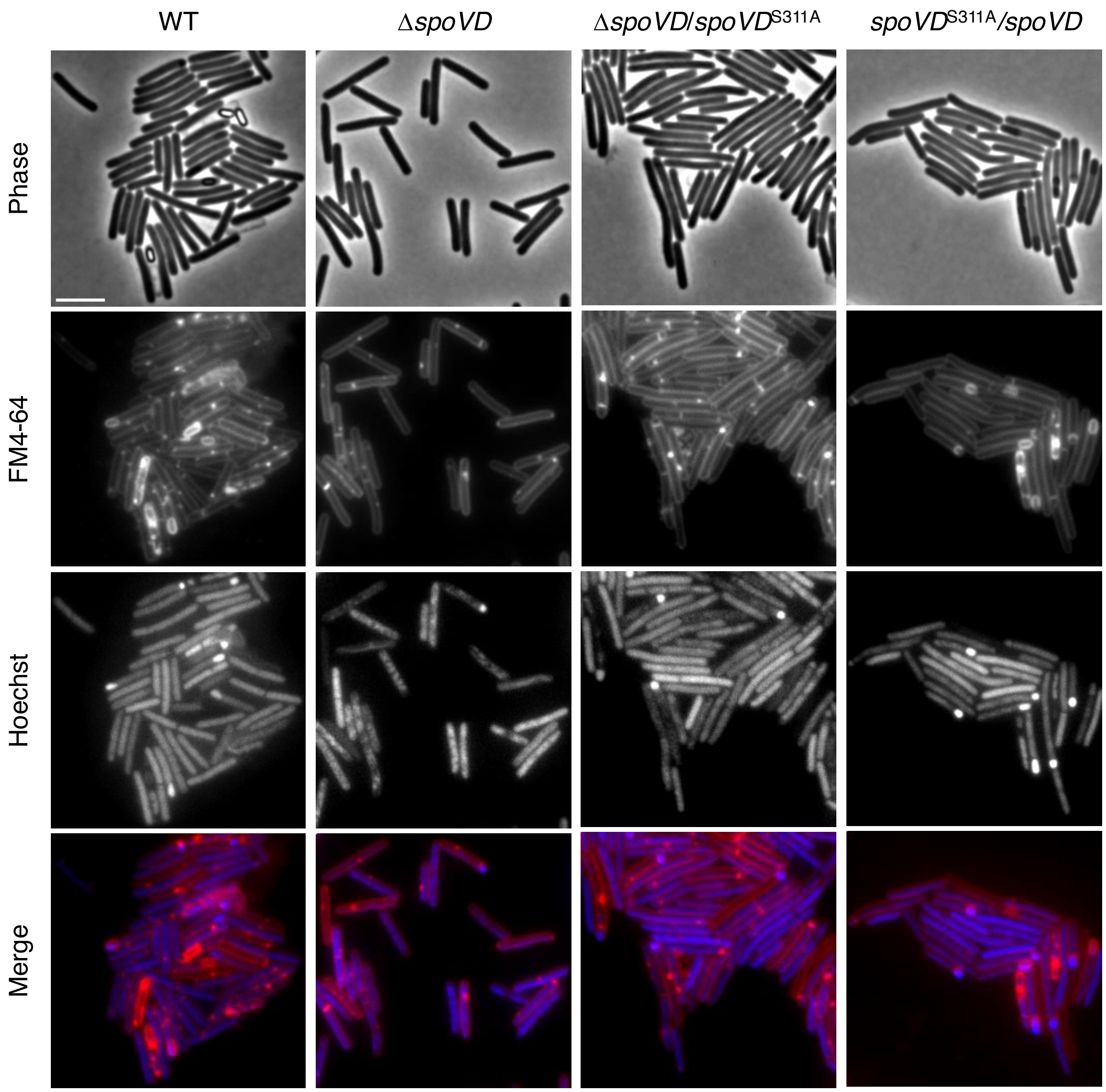

FIG S2 Cytological profiling of strains analyzed in Fig. 1. Representative phase-contrast and fluorescence micrographs of the indicated strains sampled after ~20 hours of growth on sporulation-inducing 70:30 plates. The nucleoid was stained using Hoechst, and the cell membrane was stained using FM4-64. Scale bar, 5 μm.

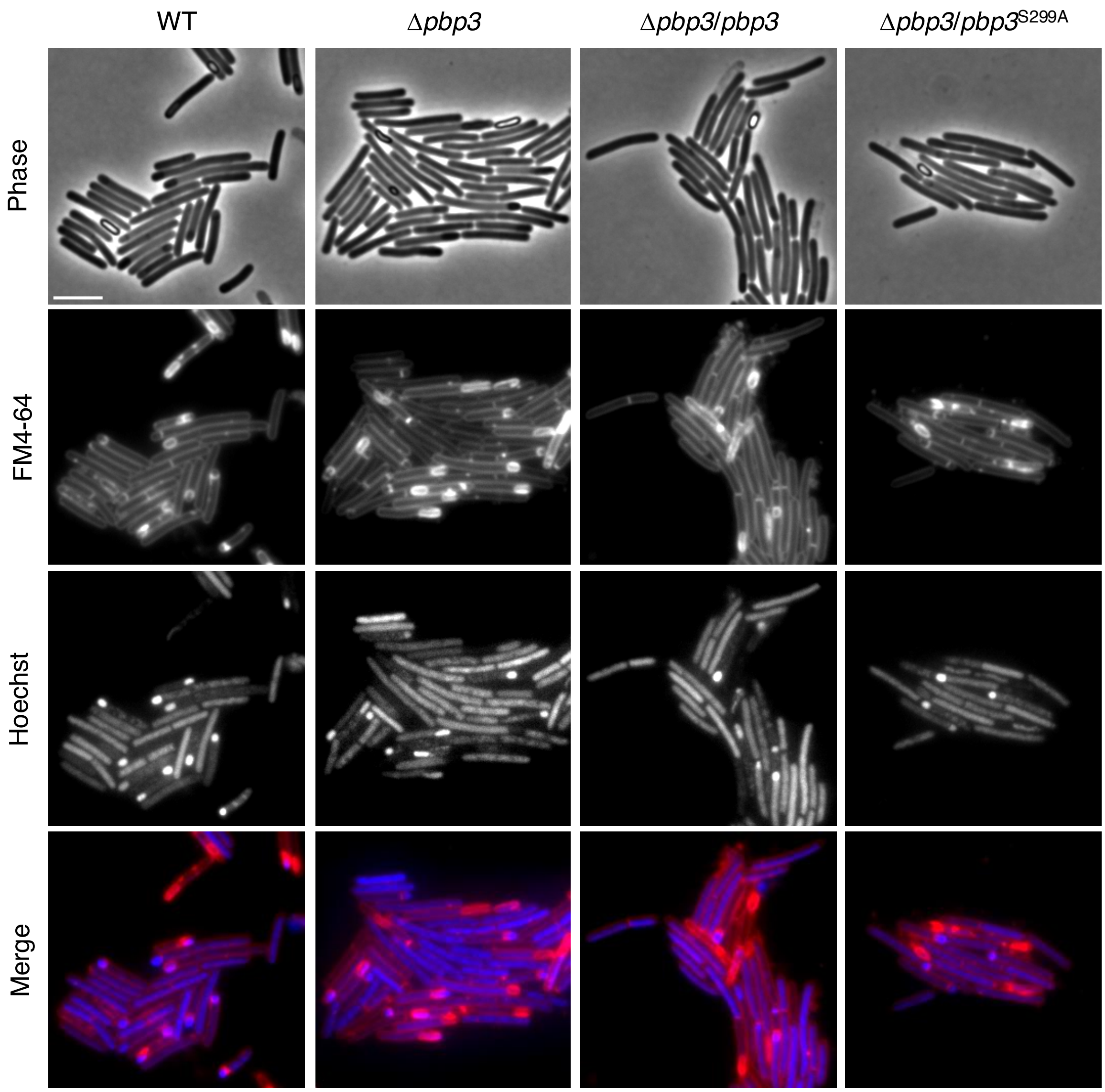

FIG S3 Cytological profiling of strains analyzed in Fig. 4. Representative phase-contrast and fluorescence micrographs of the indicated strains sampled after ~20 hours of growth on sporulation-inducing 70:30 plates. The nucleoid was stained using Hoechst, and the cell membrane was stained using FM4-64. Scale bar, 5 μm.

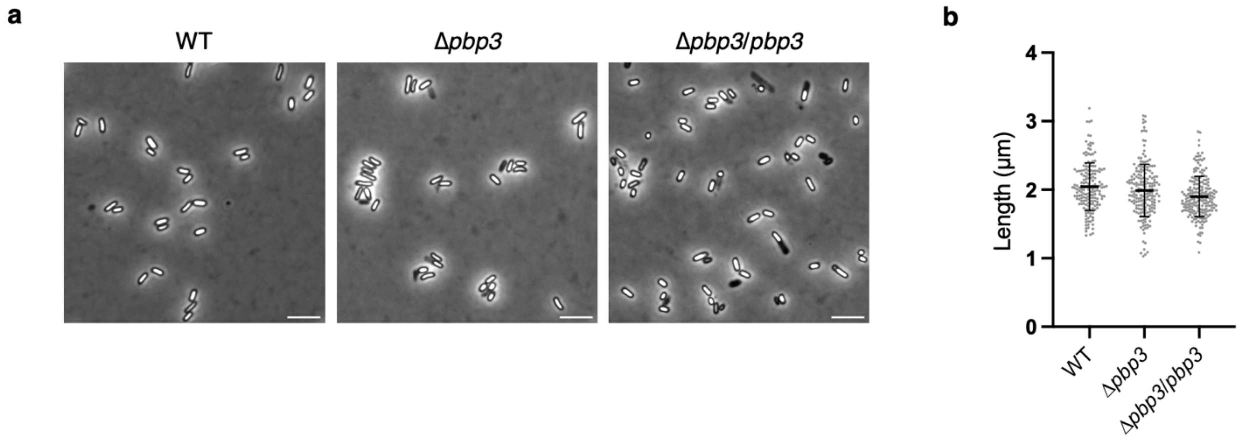

FIG S4 Similar morphologies between purified WT and Δ*pbp3* spores. (a) Phase-contrast images of purified spores from the indicated strains. Scale bar, 5 *μ*m. (b) Spore lengths measured for over 170 spores for each strain (n =1).

TABLE S1 *C. difficile* strains used in this study.

Strain number	Strain description	Description	Study
756	630Δ*erm*Δ*pyrE*	*erm*-sensitive derivate of 630 with a deletion in *pyrE*	(1)
846	630Δ*erm*-p	630Δ*erm* with *pyrE* restored in the native locus - used as the WT strain in the study	(2)
849	630Δ*erm*-p Δ*spo0A*	630Δ*erm* with a deletion of *spo0A* and *pyrE* restored	(2)
2595	630Δ*erm*-p Δ*spoVD*	630Δ*erm* with a deletion of *spoVD* (*cd630_26560*) and *pyrE* restored	(3)
2728	630Δ*erm*-p Δ*spoVD/spoVD*	630Δ*erm* with a deletion of *spoVD* (*cd630_26560*) and *pyrE* restored and complemented with *spoVD*	(3)
2734	630Δ*erm*-p *Δpbp3ΔpyrE*	630Δ*erm*Δ*pyrE* with a deletion of *pbp3* (*cd630_12290*)	(3)
2948	630Δ*erm*-p Δ*pbp3*	630Δ*erm*Δ*pbp3* with *pyrE* restored	(3)
3004	630Δ*erm*-p Δ*spoVD/spoVD*^{S311A}	630Δ*erm*Δ*spoVD* with *pyrE* restored and *spoVD*^{S311A} complemented in the *pyrE* locus	This study
3022	630Δ*erm*-p Δ*pbp3/pbp3*	630Δ*erm*Δ*pbp3* with *pyrE* restored and *pbp3* complemented in the *pyrE* locus	This study
3031	630Δ*erm*-p Δ*spoVE*	630Δ*erm*Δ*spoVE* with *pyrE* restored	(3)
3072	630Δ*erm*-p Δ*spoVE/spoVE*	630Δ*erm*Δ*spoVE* with *pyrE* restored and *spoVE* complemented in the *pyrE* locus	(3)
3326	630Δ*erm*Δ*pyrE*Δ*spoVD*Δ*pbp3*	630Δ*erm*Δ*pyrE* with a sequential deletion of *spoVD* and *pbp3*	This study
3581	630Δ*erm*-p Δ*spoVD*Δ*pbp3*	630Δ*erm*Δ*spoVD*Δ*pbp3* with *pyrE* restored	This study
4110	630Δ*erm*Δ*pyrE spoVD*^{S311A}	630Δ*erm*Δ*pyrE* with the native *spoVD* mutated (serine to alanine substitution (tct>gct) in residue 311)	This study
4348	630Δ*erm*-p *spoVD*^{S311A}	630Δ*erm*Δ*pyrE spoVD*^{S311A} with *pyrE* restored	This study
4351	630Δ*erm*-p *spoVD*^{S311A}/*spoVD*	630Δ*erm*Δ*pyrE spoVD*^{S311A} with *pyrE* restored and *spoVD* complemented in the *pyrE* locus	This study
4361	630Δ*erm*-p *spoVD*^{S311A} *Δpbp3*	630Δ*erm*Δ*pyrE spoVD*^{S311A} with deletion of *pbp3* and *pyrE* restored	This study
4390	630Δ*erm*-p *spoVD*^{S311A} *Δpbp3/pbp3*	630Δ*erm*Δ*pyrE spoVD*^{S311A}Δ*pbp3* with *pyrE* restored and *pbp3* complemented in the *pyrE* locus	This study

4432	630Δ*erm*-p Δ*pbp3/pbp3*^{S299A}	630Δ*erm*Δ*pyrE* Δ*pbp3* with *pyrE* restored and mutated *pbp3* (serine to alanine substitution (tct>gct) in residue 299) in the *pyrE* locus	This study
4441	630Δ*erm*-p Δ*pbp3*/*pbp3*$_{3XFLAG}$	630Δ*erm*Δ*pyrE* Δ*pbp3* with *pyrE* restored and *pbp3-3XFLAG* in *pyrE* locus	This study
4568	630Δ*erm*-p Δ*spoVD*/*spoVD*-$_{3XFLAG}$	630Δ*erm*Δ*pyrE* Δ*spoVD* with *pyrE* restored and *spoVD-3XFLAG* in *pyrE* locus	This study
4574	630Δ*erm*-p *spoVD*^{S311A} Δ*pbp3/pbp3*^{S299A}	630Δ*erm*Δ*pyrE* *spoVD*^{S311A}Δ*pbp3* with *pyrE* restored and *pbp3* ^{S299A} complemented in the *pyrE* locus	This study
4623	630Δ*erm*-p *spoVD*^{S311A} Δ*spoVE*	630Δ*erm*Δ*pyrE* *spoVD*^{S311A} with deletion of *spoVE* and *pyrE* restored	This study
4626	630Δ*erm*-p *spoVD*^{S311A} Δ*spoVE/spoVE*	630Δ*erm*Δ*pyrE* *spoVD*^{S311A}Δ*spoVE* with *pyrE* restored and *spoVE* complemented in the *pyrE* locus	This study
4759	630Δ*erm*-p P$_{tet}$-*mScarlet-I3-pbp1*	630Δ*erm*Δ*pyrE* with *pyrE* restored with aTc-inducible mScarlet-I3 fusion to the N-terminus of PBP1	(4)
5270	630Δ*erm*-p P$_{tet}$-*mScarlet-I3-pbp3*	630Δ*erm*Δ*pyrE* with *pyrE* restored with aTc-inducible mScarlet-I3 fusion to the N-terminus of PBP3	(4)
5625	630Δ*erm*-p Δ*pbp3* P$_{tet}$-*mScarlet-I3-pbp1*	630Δ*erm*Δ*pyrE* Δ*pbp3* with *pyrE* restored with aTc-inducible mScarlet-I3 fusion to the N-terminus of PBP1	This study
5785	630Δ*erm*-p Δ*spoVD* P$_{tet}$-*mScarlet-I3-pbp1*	630Δ*erm*Δ*pyrE* Δ*spoVD* with *pyrE* restored with aTc-inducible mScarlet-I3 fusion to the N-terminus of PBP1	This study
5789	630Δ*erm*-p Δ*spoVD*Δ*pbp3* P$_{tet}$-*mScarlet-I3-pbp1*	630Δ*erm*Δ*pyrE* Δ*spoVD*Δ*pbp3* with *pyrE* restored with aTc-inducible mScarlet-I3 fusion to the N-terminus of PBP1	This study
5791	630Δ*erm*-p Δ*spoVD* P$_{tet}$-*mScarlet-I3-pbp3*	630Δ*erm*Δ*pyrE* Δ*spoVD* with *pyrE* restored with aTc-inducible mScarlet-I3 fusion to the N-terminus of PBP3	This study
5627	630Δ*erm*-p Δ*spoVD* Δ*pbp3/spoVD-FLAG*$_{3}$	630Δ*erm* Δ*spoVD* Δ*pbp3* with *pyrE* restored and complemented with *spoVD-FLAG*$_{3}$	This study

TABLE S2 *E. coli* strains used in this study.

Strain number	Strain description	Relevant genotype or link to annotated plasmid sequence	Source
41	DH5α	F^{-} Φ80*lacZ*ΔM15 Δ(*lacZYA-argF*) U169 *recA1 endA1 hsdR17* (rK^{-}, mK^{+}) *phoA supE44* λ– *thi-1 gyrA96 relA1*	D. Cameron
531	HB101	F^{-} *mcrB mrr hsdS20*(rB^{-}mB^{-}) *recA13 leuB6 ara-13 proA2 lavYI galK2 xyl-6 mtl-1 rpsL20* carrying pRK24	C. Ellermeier
1218	BTH101	F^{-}, *cya-99, araD139, galE15, galK16, rpsL1* (Str^{r})*, hsdR2, mcrA1, mcrB1*	Euromedex
1452	XL1-Blue	*recA1 endA1 gyrA96 thi-1 hsdR17 supE44 relA1 lac* carrying F *proAB lacIqZΔM15 Tn10* (Tet^{r})	C. Huston
1539	DH5α / pMTL-YN3	pMTL-YN3 in DH5α	(1)
1662	HB101/pRK24/ pMTL-YN1C	pMTL-YN1C in HB101/pRK24	(1)
2469	HB101/pRK24/ pMTL-YN3 *Δpbp3*	https://benchling.com/s/seq-ZXq5K09mLbsPHse4YNTa	(3)
2475	HB101/pRK24/ pMTL-YN1C-*spoVD*	https://benchling.com/s/seq-rB133tLjJlc7rJJXQtZs	(3)
2627	HB101/pRK24/ pMTL-YN1C-*spoVD^{S311A}*	https://benchling.com/s/seq-RlSZsqjrdgU9pUnl101G	This study
2631	HB101/pRK24/ pMTL-YN1C-*pbp3*	https://benchling.com/s/seq-Y7cfuxWOsm45Pz7Q8veh	This study
2672	HB101/pRK24/ pMTL-YN1C-*spoVE*	https://benchling.com/s/seq-J2BDth9Xje1Ry0RYL8yY	(3)
3354	HB101/pRK24/ pMTL-YN3-*spoVD^{S311A}*	https://benchling.com/s/seq-RlSZsqjrdgU9pUnl101G	This study
3568	BL21(DE3) pET28a-*pbp1*$_{Δ1-78}$	https://benchling.com/s/seq-H4Q8cuaGUBznJVmcUxWr	This study
3611	HB101/pRK24/ pMTL-YN1C-*spoVD*^{3XFLAG}	https://benchling.com/s/seq-8ozjvbJl1aFC4yvUZ93R	This study
3697	HB101/pRK24/ pMTL-YN1C *pbp3^{S299A}*	https://benchling.com/s/seq-W0iG7JIhBgkWkIY30JSn	This study
3699	HB101/pRK24/ pMTL-YN1C *pbp3*^{3XFLAG}	https://benchling.com/s/seq-84uLyNFxTAf0F0dmy7W7	This study
3709	BL21(DE3) pET28a *pbp3*$_{ΔTM}$-His$_{6}$	https://benchling.com/s/seq-Na7IhwYzfBMC3cgvWEDq	This study
3151	XL1-Blue pUT18C-*spoVD*	https://benchling.com/s/seq-CoDkpnu9UjIw1NGMf7K3	(3)
3152	XL1-Blue pKT25-*spoVD*	https://benchling.com/s/seq-WE1eUEvUa9oPe1SHOONt	(3)
3153	XL1-Blue pUT18C-*spoVE*	https://benchling.com/s/seq-J6xY0qAz4gLUCvIsvPtr	(3)
3154	XL1-Blue pKT25-*spoVE*	https://benchling.com/s/seq-q0Hi3dYXXUh38AIJmwLo	(3)
3155	XL1-Blue pUT18C-*ftsL*	https://benchling.com/s/seq-bmQB9ktQ33MqNQkBLm55	(3)
3156	XL1-Blue pKT25-*ftsL*	https://benchling.com/s/seq-EMRCycRB9UdqQCbeX9jT	(3)
3157	XL1-Blue pUT18C-*ftsQ*	https://benchling.com/s/seq-iL0HyX5G3dtQADBqOFfK	(3)

3158	XL1-Blue pKT25-*ftsQ*	https://benchling.com/s/seq-JEX75QVog9sFurh310Wa	(3)
3159	XL1-Blue pUT18C-*ftsB*	https://benchling.com/s/seq-ZXq5K09mLbsPHse4YNTa	(3)
3160	XL1-Blue pKT25-*ftsB*	https://benchling.com/s/seq-rB133tLjJlc7rJJXQtZs	(3)
3161	XL1-Blue pUT18C-*pbp3*	https://benchling.com/s/seq-RlSZsqjrdgU9pUnI101G	This study
3162	XL1-Blue pKT25-*pbp3*	https://benchling.com/s/seq-Y7cfuxWOsm45Pz7Q8veh	This study
3339	XL1-Blue pUT18C-*pbp1*	https://benchling.com/s/seq-J2BDth9Xje1Ry0RYL8yY	This study
3340	XL1-Blue pKT25-*pbp1*	https://benchling.com/s/seq-RlSZsqjrdgU9pUnI101G	This study
3341	XL1-Blue pUT18C-*pbp2*	https://benchling.com/s/seq-H4Q8cuaGUBznJVmcUxWr	This study
3342	XL1-Blue pKT25-*pbp2*	https://benchling.com/s/seq-8ozjvbJl1aFC4yvUZ93R	This study
3343	XL1-Blue pUT18C-*rodA*	https://benchling.com/s/seq-W0iG7JIhBgkWkIY30JSn	This study
3344	XL1-Blue pKT25-*rodA*	https://benchling.com/s/seq-84uLyNFxTAf0F0dmy7W7	This study
3747	HB101/pRK24/ pMTL-YN1C-P_{tet}-*mScarlet-I3-pbp1*$_{im}$	https://benchling.com/s/seq-Na7IhwYzfBMC3cgvWEDq	(4)
4390	HB101/pRK24/ pMTL-YN1C-P_{tet}-*mScarlet-I3-pbp3*	https://benchling.com/s/seq-CoDkpnu9UjIw1NGMf7K3	(4)

References

1. Ng YK, Ehsaan M, Philip S, Collery MM, Janoir C, Collignon A, Cartman ST, Minton NP. 2013. Expanding the Repertoire of Gene Tools for Precise Manipulation of the *Clostridium difficile* Genome: Allelic Exchange Using pyrE Alleles. PLoS One 8:e56051.
2. Donnelly ML, Li W, Li Y, Hinkel L, Setlow P, Shen A. 2017. A *Clostridium difficile*-Specific, Gel-Forming Protein Required for Optimal Spore Germination. mBio 8:10.1128/mbio.02085-16.
3. Shrestha S, Taib N, Gribaldo S, Shen A. 2023. Diversification of division mechanisms in endospore-forming bacteria revealed by analyses of peptidoglycan synthesis in *Clostridioides difficile*. Nat Commun 14:7975.
4. Harrison GA, Shen A. 2025. Molecular dissection of Class A PBP function uncovers novel features of the non-canonical *Clostridioides difficile* divisome complex. PLoS Genet 21:e1011746.
